# A cross-sectional study on the correlation between internal cerebral vein asymmetry and hemorrhagic transformation following endovascular thrombectomy

**DOI:** 10.3389/fneur.2024.1465481

**Published:** 2025-01-07

**Authors:** Kunxin Lin, Wenlong Zhao, Quanhong Wu, Yiru Zheng, Bo Yang, Ying Fu, Ning Wang, Ling Fang

**Affiliations:** ^1^Department of Neurology and Institute of Neurology of First Affiliated Hospital, Institute of Neuroscience, Fujian Key Laboratory of Molecular Neurology, Fujian Medical University, Fuzhou, China; ^2^Department of Neurology, National Regional Medical Center, Binhai Campus of the First Affiliated Hospital, Fujian Medical University, Fuzhou, China; ^3^Department of Radiology, First Affiliated Hospital of Fujian Medical University, Fuzhou, China

**Keywords:** internal cerebral veins, hemorrhagic transformation, acute ischemic stroke, endovascular thrombectomy, large vessel occlusion

## Abstract

**Introduction:**

Hemorrhagic transformation (HT) is a severe complication in patients with acute ischemic stroke due to large vessel occlusion (AIS-LVO) after endovascular treatment (EVT). We hypothesize that asymmetry of the internal cerebral veins (ICVs) on baseline CT angiogram (CTA) may serve as an adjunctive predictor of HT.

**Methods:**

We conducted a study on consecutive AIS-LVO patients from November 2020 to April 2022. These patients had anterior circulation occlusions and were treated with EVT. Asymmetrical ICVs were assessed using CTA and defined as hypodensity (reduced opacification) on the ipsilateral side of occlusion compared to the contralateral side. The primary outcome was HT, defined as hemorrhage within the ischemic territory. This was evaluated using follow-up imaging (CT scan or magnetic resonance imaging) performed 48 h post-EVT. HT was classified into four subtypes based on the European Cooperative Acute Stroke Study-II criteria.

**Results:**

A total of 126 patients were included, with an HT rate of 49.2% (62/126). ICV asymmetry was observed in 54.0% (68/126) of patients. The ICV asymmetry group exhibited a significantly higher risk of parenchymatous hematoma-type HT (33.8% vs. 15.5%, *p* = 0.019) and symptomatic intracerebral hemorrhage (sICH) (23.5% vs. 5.2%, *p* = 0.004). In multivariate logistic regression, ICV asymmetry (OR 3.809, 95% CI 1.582–9.171), baseline Alberta Stroke Program Early CT Score (OR 0.771, 95% CI 0.608–0.978), intravenous recombinant tissue plasminogen activator (OR 2.847, 95% CI 1.098–2.7.385), and poor collateral circulation (OR 3.998, 95% CI 1.572–10.169) were identified as independent risk factors of HT.

**Conclusion:**

ICV asymmetry, likely resulting from impaired autoregulation or tissue micro-perfusion hampering cerebral blood flow (CBF), is a novel radiological sign that independently predicts HT. It is associated with a higher risk of sICH in AIS-LVO patients after EVT. Further research is warranted to validate these findings.

## 1 Introduction

Endovascular thrombectomy (EVT) has become the standard of care for patients with acute ischemic stroke caused by large vessel occlusion (AIS-LVO) in recent years (1). The time window for EVT was extended to 24 h based on findings from the DAWN and DIFFUSE-3 trials, which utilized diffusion-weighted imaging to better understand stroke evolution. As a result, more AIS-LVO patients could be treated. However, severe complications, particularly hemorrhagic transformation (HT), pose significant challenges to its widespread application. Intracranial HT is a common and serious complication often associated with poor outcomes (2). Reported incidence rates in clinical trials are as high as 49.5% (3). Given its clinical impact, identifying risk factors associated with HT is critically important to help clinicians make informed treatment decisions and improve patient prognosis.

Arterial collaterals only reflect the arterial portion of the microcirculation and are not a reliable indicator of cerebral perfusion (4). Conversely, venous drainage is generally a better reflection of cerebral microcirculation and perfusion. Unobstructed venous drainage indicates adequate cerebral blood flow through brain tissue (5).

The internal cerebral veins (ICVs), located near the interventricular foramen, are responsible for draining venous drainage from the deep structures of the cerebral hemisphere. These veins converge to form the great vein of Galen, which subsequently drains into the straight sinus (6). In cases of anterior large vessel occlusion, patients may experience reduced blood flow in deep brain tissues, potentially leading to hypo-opacification of the ipsilateral ICV, a phenomenon referred to as ICV asymmetry.

Recent studies have shown that ICV asymmetry is associated with poor functional outcomes (7, 8). Favorable venous outflow profiles, on the other hand, are associated with reduced early edema progression and improved functional outcomes in AIS-LVO patients undergoing EVT (9). However, limited research has specifically explored the relationship between ICV asymmetry and HT after EVT.

We hypothesized that ICV asymmetry is associated with HT in AIS-LVO patients who underwent EVT. We verified this hypothesis by determining ICV symmetry using baseline CTA images and evaluated HT through 48-h follow-up head non-contrast CT (NCCT) or magnetic resonance imaging (MRI) after EVT.

## 2 Materials and methods

### 2.1 Study population

In an ongoing study (ClinicalTrials.gov Identifier NCT 04637074), we prospectively collected demographic characteristics and clinical and imaging data of patients with AIS-LVO who underwent EVT between November 2020 and April 2022. Following this data collection, a retrospective analysis of all patients was performed to analyze the relationship between ICV asymmetry and HT. The data collection process was approved by the ethics committee, and written informed consent was obtained from all patients.

The inclusion criteria were as follows: (1) age ≥ 18 years old; (2) EVT < 24 h after stroke onset; and (3) large vessel occlusion of the anterior circulation (internal carotid artery or first (M1) or second (M2) segment of the middle cerebral artery) determined by CT angiography (CTA) examination. The exclusion criteria were as follows: (1) subarachnoid hemorrhage or intracranial hemorrhage determined by a baseline NCCT scan; (2) poor CTA image quality resulting from motion artifacts and inability to assess ICVs asymmetry; (3) absence of follow-up head NCCT/MRI after EVT; and (4) history of iodine allergy.

### 2.2 Imaging acquisition and treatment

All patients underwent head NCCT or CTA performed on a 64-section CT scanner (Toshiba Medical Systems, Nasu, Japan). NCCT was performed (120 kV; 300 mA; section thickness, 5 mm) from the foramen magnum to the vertex. In our study, helical CTA was performed using a bolus tracking method to determine the optimal timing for image acquisition. After administering 40 mL of an iodinated contrast medium (iopamidol 370, Braccosine, Shanghai, China) at a flow rate of 5.0 mL/s, followed by a 35-mL saline flush using a two-channel high-pressure injector, helical CTA was conducted from the foramen magnum to the vertex with a section thickness of 0.625 mm.

All patients underwent follow-up MRI using a 1.5-T scanner (Signa Excite; GE Medical Systems, Milwaukee, WI, United States) with an 8-channel head coil. The MR protocol included a routine head scan including T1-weighted imaging (T1WI), T2-weighted imaging (T2WI), diffusion-weighted imaging (DWI), and T2-weighted fluid-attenuated inversion recovery (FLAIR).

All patients who presented to our hospital underwent an urgent NCCT of the brain to exclude intracranial hemorrhage and were treated with intravenous recombinant tissue plasminogen activator (IV-rtPA) if they met the criteria. Consequently, EVT was usually performed if a large vessel occlusion was confirmed by CTA.

### 2.3 Imaging analysis

Imaging data were independently reviewed by two neuroradiologists, each with over 5 years of experience in stroke imaging, who were blinded to all patients’ clinical and outcome data. Any disagreements between the two observers were resolved by a senior medical radiologist. All patients underwent non-contrast head CT (NCCT) and CT angiography (CTA), followed by NCCT or MRI 48 h post-EVT.

The Alberta Stroke Program Early CT Score (ASPECTS) (10) was used to assess the early ischemic burden on baseline NCCT imaging. Baseline CTA source images were used to assess ICV opacification. We assessed the degree of ICV opacification using maximum intensity projection (MIP) sequences on axial sections. ICV asymmetry was defined as lesser opacification (hypodensity) of the ICV on the ipsilateral ischemic hemisphere compared to the contralateral ICV. Conversely, if the degree of opacification was equal or more than the contralateral ICV, the ICVs are considered symmetric.

Collateral circulation was graded on pre-recanalization angiograms using the American Society of Interventional and Therapeutic Neuroradiology/Society of Interventional Radiology (ASITN/SIR) guidelines, with grades 3–4 classified as good collaterals. Successful recanalization of the occluded artery after EVT was defined as a modified Thrombolysis in Cerebral Infarction (mTICI) grade 2b/3 on final angiography. Any disagreement was resolved through consensus coordination.

### 2.4 Clinical data collection

We prospectively collected all the variables, such as demographic characteristics, vascular risk factors (hypertension, hyperlipidemia, diabetes mellitus, smoking, and atrial fibrillation), previous medication history (antiplatelets and anticoagulants), blood pressure, baseline stroke scale (e.g., National Institutes of Health Stroke Scale [NIHSS]), imaging data, procedural details, time-metric data, and prognosis outcome (e.g., modified Rankin Scale [mRS] at 90 days). The probable stroke etiology was categorized according to the Trial of ORG 10172 in Acute Stroke Treatment (TOAST) criteria (11).

### 2.5 Outcomes

HT refers to hemorrhagic transformation within the ischemic region, evaluated on follow-up imaging after EVT and classified into four subtypes based on the European Cooperative Acute Stroke Study-II (ECASS II) (12). In cases of inconsistency between the two ratings, the severity rating takes precedence. Symptomatic intracranial hemorrhage (sICH) is defined as neurological deterioration (resulting in an increase of ≥4 points on the NIHSS within 48 h) accompanied by evidence of intracranial hemorrhage on brain CT or MRI performed 48 h after treatment. The modified Rankin scale (mRS) score after 3 months was used to evaluate the patient’s clinical outcomes, and patients with an mRS score of 3 to 6 were classified as having a poor prognosis.

### 2.6 Statistical analysis

Normally distributed continuous variables were expressed as mean ± standard deviation (SD), and non-normally distributed variables were described as median (interquartile range, IQR). Categorical variables were expressed as frequency (percentage). The chi-squared test was used to compare categorical variables between patients with HT and those with non-HT. The Mann–Whitney U test was employed to analyze non-normally distributed variables between the two groups. A Student unpaired t-test was performed for normally distributed variables between the two groups. Multivariable binary logistic regression was performed with factors associated with HT with *p* < 0.1. Collinearity among predictors was evaluated using the Spearman correlation coefficient and the variance inflation factor (VIF). Interrater reliability of ICV status was tested by *κ* statistics. Statistical significance was set as a probability value of <0.05, and all reported results were two-sided. Statistical analyses were conducted using SPSS Version 25.0 (SPSS Inc., Chicago, IL, United States).

## 3 Results

### 3.1 Baseline characteristics of patients

**Figure 1:**
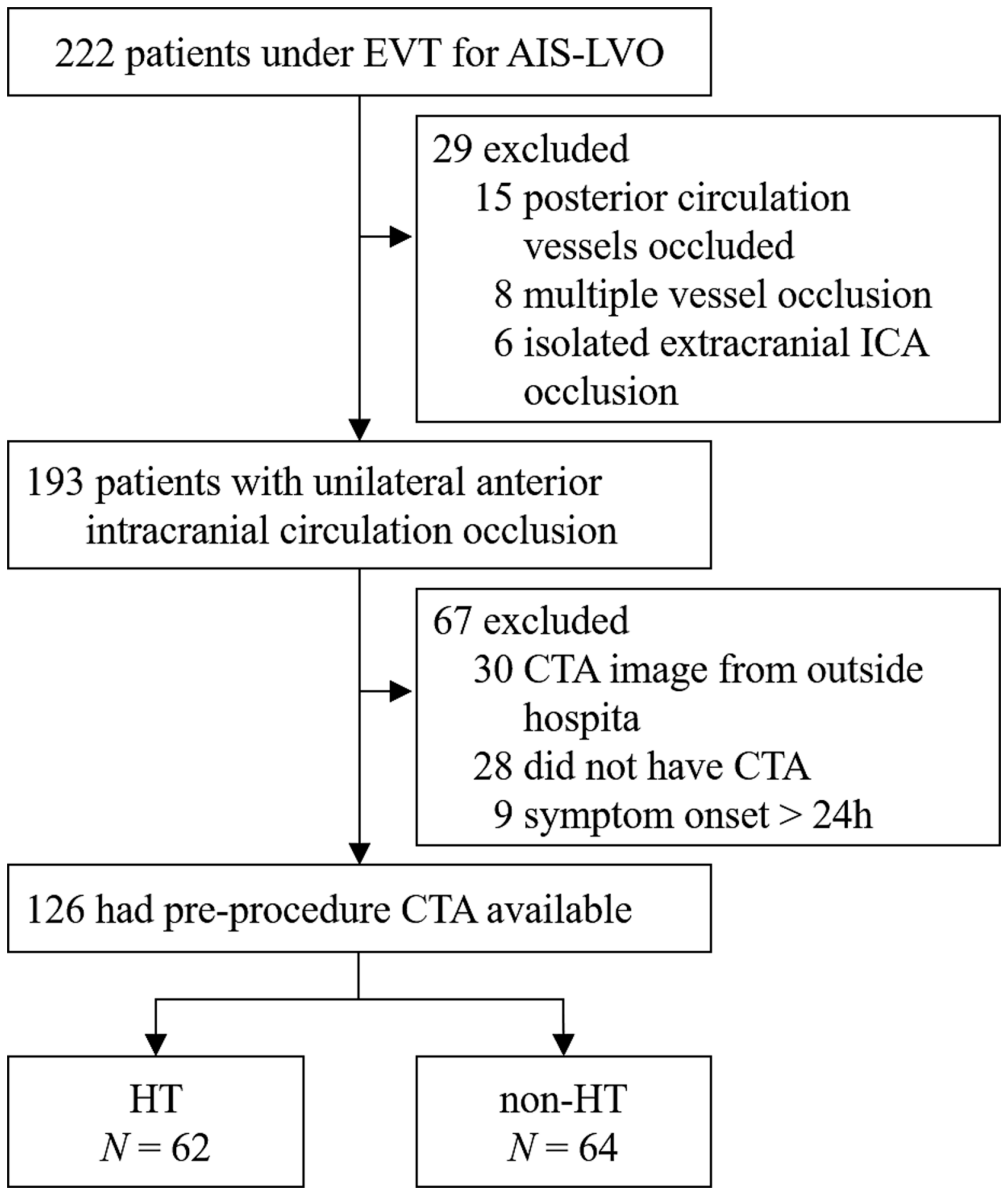
A flow chart of patient selection. EVT, endovascular treatment; AIS-LVO, acute ischemic stroke due to large vessel occlusion; CTA, computed tomography angiography; MCA, middle cerebral artery; ICA, internal carotid artery; HT, hemorrhagic transformation.

A total number of 126 patients met the inclusion criteria (Figure 1). Overall, the HT rate was 49.2% (62/126). Of the 126 patients, 84 (66.7%) had hypertension, 31 (24.6%) had diabetes mellitus, 51 (40.5%) had atrial fibrillation, and only 9 (7.1%) had hyperlipidemia. Based on the TOAST classification, the etiology of stroke was large-artery atherosclerosis in 45 (35.7%) patients and cardiogenic embolism in 51 (40.5%) patients. A total of 29 (23.0%) patients had an undetermined reason for stroke. All baseline demographic and clinical characteristics are presented in Table 1. The median time from symptom onset to puncture was 511.50 min (range 403.5–758.00 min), while the median time from puncture to recanalization was 56.0 min (range 44.75–74.5 min). More details are shown in Table 2.

**Table 1:** Baseline characteristics of HT and non-HT group.

Variables	HT (*N* = 62)	Non-HT (*N* = 64)	*p*-Value
Age (years), median (IQR)	69 (56–74)	70 (59–78)	0.323
Female, *N* (%)	21 (33.9)	20 (31.3)	0.754
Medical history			
Hypertension, *N* (%)	41 (66.1)	43 (67.2)	0.900
Diabetes mellitus, *N* (%)	16 (25.8)	15 (23.4)	0.758
Hyperlipidemia, *N* (%)	5 (8.1)	4 (6.3)	0.742
Atrial fibrillation, *N* (%)	28 (45.2)	23 (35.9)	0.292
Blood glucose (mmol/L), median (IQR)	7.20 (6.10–10.04)	7.20 (5.95–8.31)	0.249
Smoking, *N* (%)	17 (27.4)	19 (29.7)	0.778
Previous stroke, *N* (%)	9 (14.5)	9 (14.1)	0.942
Anticoagulation treatment, *N* (%)	7 (11,3)	4 (6.3)	0.316
Antiplatelet treatment, *N* (%)	4 (6.5)	8 (12.5)	0.248
Stroke cause, *N* (%)			0.058
Cardioembolism	31 (50.0)	21 (32.8)	
Large-artery atherosclerosis	16 (25.8)	29 (45.3)	
Undetermined etiology	15 (24.2)	14 (21.9)	
Stroke presentation details			
Presentation NIHSS score, median (IQR)	17 (12–20)	15 (8–18)	0.036^*^
SBP (mm Hg), mean (SD)	149 (25)	144 (21)	0.053
DBP (mm Hg), mean (SD)	88 (19)	83 (16)	0.120
Time from onset to admission imaging (min) median (IQR)	368 (264–510)	377 (254–591)	0.880
ASPECTS, median (IQR)	8 (7–9)	9 (8–10)	0.006^*^
ICVs asymmetry, *N* (%)	45 (72.6)	23 (35.9)	<0.001^*^

^*^p < 0.05 indicates statistical significance.

ASPECTS, Alberta Stroke Program Early CT Score; DBP, diastolic blood pressure; HT, hemorrhagic transformation; ICVs, internal cerebral veins; NIHSS, National Institutes of Health Stroke Scale; SBP, systolic blood pressure.

**Table 2:** Procedural and outcome characteristics of HT and non-HT groups.

Demographics	HT (*N* = 62)	Non-HT (*N* = 64)	*p*-Value
IV-rtPA, *N* (%)	23 (37.1)	12 (17.6)	0.022^*^
Onset to puncture (min), median (IQR)	538 (383–727)	510 (407–789)	0.634
admission imaging to puncture time, median (IQR)	130 (100–166)	133 (97–182)	0.638
Puncture to recanalization (min), median (IQR)	55 (45–75)	58 (44–75)	0.946
Location of vessel occlusion, *N* (%)			0.036^*^
Internal carotid artery	28 (45.2)	15 (23.4)	
MCA 1 segment	31 (50.0)	44 (68.8)	
MCA 2 segment	3 (4.8)	5 (7.8)	
Collateral score, *N* (%)			<0.001^*^
Poor collaterals (ASITN/SIR <3)	48 (77.4)	26 (40.6)	
Good collaterals (ASITN/SIR ≥3)	14 (22.6)	38 (59.4)	
Passes of the thrombectomy device, *N* (%)			0.060
≤ 2	53 (85.5)	61 (95.3)	
> 2	9 (14.5)	3 (4.7)	
Recanalization after thrombectomy (mTICI 2b-3), *N* (%)	61 (98.4)	62 (96.9)	1.000
Emergent angioplasty, *N* (%)			
Balloon angioplasty	6 (9.7)	9 (14.1)	0.447
Rescue stenting	5 (8.1)	4 (6.3)	0.742
Poor outcome (mRS score, 3–6), *N* (%)	47 (75.8)	33 (51.2)	0.005^*^

^*^p < 0.05 indicates statistical significance.

ASITN/SIR, American Society of Interventional and Therapeutic Neuroradiology/Society of Interventional Radiology; IV-rtPA, intravenous recombinant tissue plasminogen activator; MCA, middle cerebral artery; mTICI, modified thrombolysis in cerebral infarction; mRS, modified Rankin Scale.

### 3.2 Association between ICV asymmetry and the risk of HT

In the univariate analysis, the factors associated with HT compared with the non-HT group were ICV asymmetry (45% versus 23%, respectively, *p* < 0.001), baseline NIHSS score (median, 17 vs. 15, respectively, *p* = 0,036), baseline ASPECTS (mean, 8 versus 9, respectively; *p* = 0.006), IV-rtPA (23% vs. 12%, respectively; *p* = 0.022), and poor collateral circulation (48% vs. 23%, respectively, *p* < 0.001). These findings are summarized in Tables 1, 2. Compared with the ICV symmetry group, the ICV asymmetry group exhibited a higher risk of any HT (66.2% vs. 29.3%, *p* < 0.001), parenchymatous hematoma (PH) type HT (33.8% vs. 15.5%, *p* = 0.019), and sICH (23.5% vs. 5.2%, *p* = 0.004) (Table 3).

**Table 3:** Hemorrhagic transformation and 90d outcomes depending on ICV asymmetry group.

Outcomes	ICVs asymmetry (*N* = 68)	ICVs symmetry (*N* = 58)	*p*-Value
Any HT	45 (66.2%)	17 (29.3%)	<0.001^*^
PH type	23 (33.8%)	9 (15.5%)	0.019^*^
sICH	16 (23.5%)	3 (5.2%)	0.004^*^
90d mRS >2	52 (65.0%)	28 (35%)	0.001^*^

^*^p < 0.05 indicates statistical significance.

HT, hemorrhagic transformation; mRS, modified Rankin scale; PH, parenchymal hematoma; sICH, symptomatic intracranial hemorrhage.

**Figure 2:**
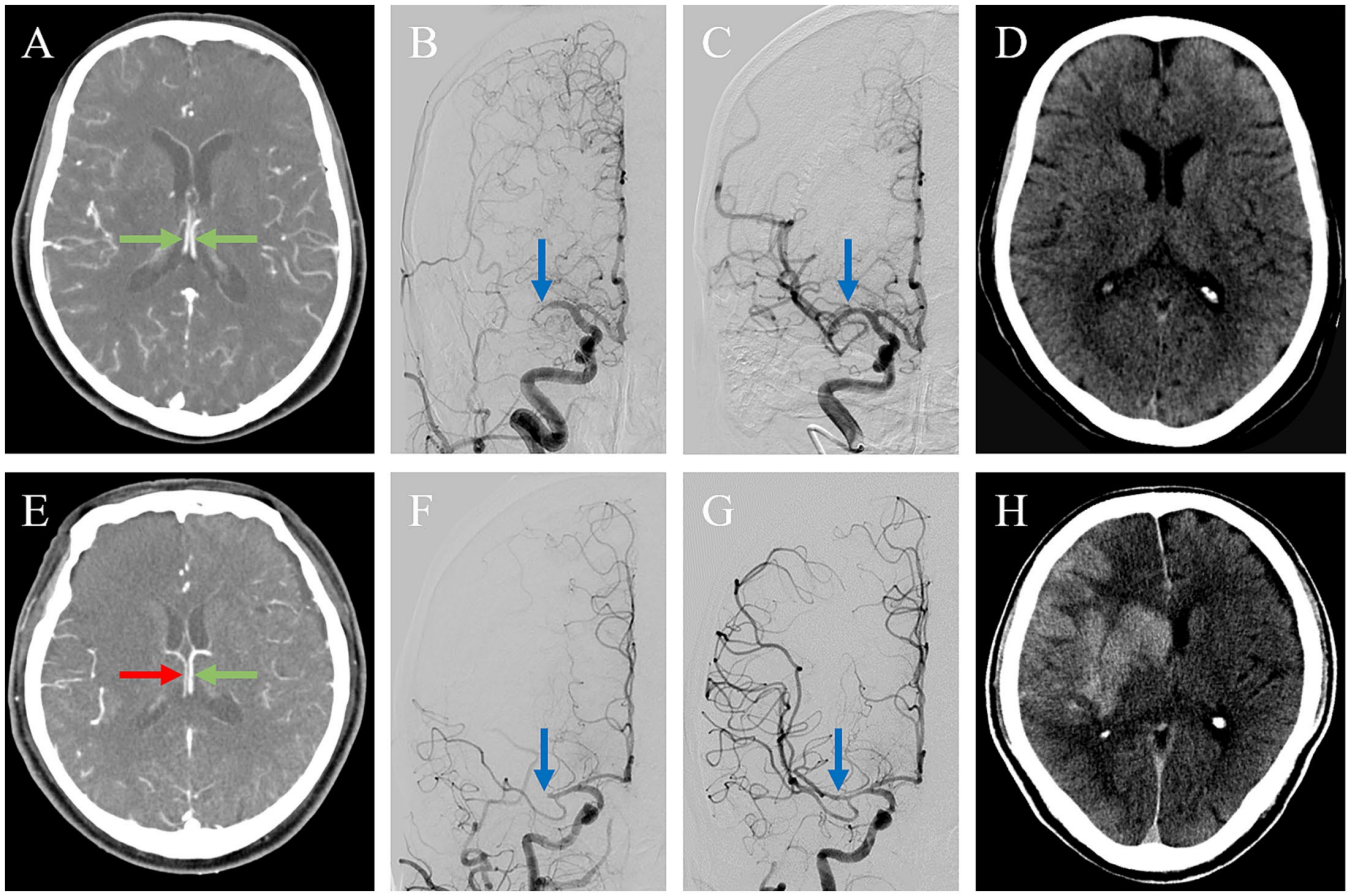
Association between ICV asymmetry and HT. Two patients with acute ischemic stroke due to occlusion of the M1 segment of the right middle cerebral artery (MCA), **(A–D)** 1 with internal cerebral veins (ICVs) symmetry and (E-H) 1 with ICVs asymmetry. In the first patient, axial maximum intensity projection imaging shows the ICVs running parallel to each other and symmetrical opacification (green arrows) **(A)**. After endovascular thrombectomy, the initial proximal MCA occlusion (blue arrow) **(B)** was successfully reperfused with a modified Thrombolysis in Cerebral Infarction (mTICI) of 3(blue arrow) **(C)**. The non-contrast CT on 24 h showed a small acute infarct without hemorrhagic transformation. The second patient exhibited a poor ICV profile in the right hemisphere [**(E)**, red arrow] and good opacification in the left (green arrow). The thrombus on the right proximal MCA (blue arrow) **(F)** was successfully reperfused with an mTICI of 3 (blue arrow) **(G)**. The non-contrast CT on 24 h showed hemorrhagic transformation with a cerebral hernia **(H)**.

**Figure 3:**
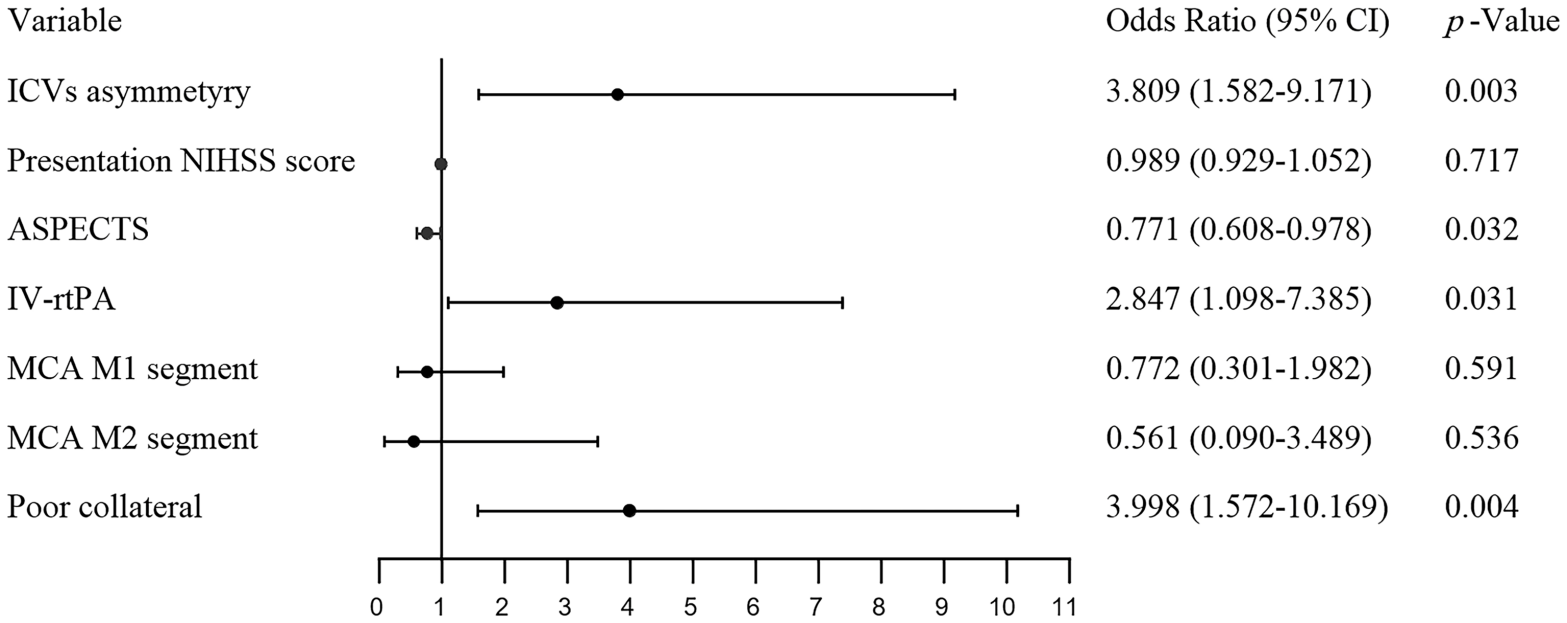
Forest plot visualizing the results of the multivariate regression analysis. ICVs, internal cerebral veins; NIHSS, National Institutes of Health stroke Scale; ASPECTS, Alberta Stroke Program Early CT Score; IV-rtPA, intravenous recombinant tissue plasminogen activator; MCA, middle cerebral artery.

The multivariate logistic regression model adjusted for the following factors: ICV asymmetry, presentation NIHSS score, ASPECTS, IV-rtPA, location of vessel occlusion, and poor collateral (ASITN/SIR <3). The analysis revealed that ICV asymmetry was associated with a higher risk of HT (OR 3.809, 95% CI 1.582–9.171), as illustrated in Figure 2. Baseline ASPECTS (OR 0.771, 95% CI 0.608–0.978) was also linked with a lower risk of HT. IV-rtPA (OR 2.847, 95% CI 1.098–2.7.385) and poor collateral circulation (OR 3.998, 95% CI 1.572–10.169) are also independent risk factors of HT. Detailed results are organized in Table 4 and Figure 3. The Spearman correlation coefficient between ICV asymmetry and collateral circulation was −0.224 (*p* = 0.012). Collinearity diagnostics showed a VIF of 1.052 and a tolerance of 0.950, indicating no significant collinearity. Interrater reliability for ICV asymmetry on baseline CTA source images was substantial (*κ* = 0.86, 95% CI 0.74 to 0.97). For HT determination, the interrater reliability was excellent (κ = 0.92, 95% CI 0.85 to 0.99), while for HT classification, it was also high (κ = 0.90, 95% CI 0.84 to 0.97). These results indicate strong and consistent agreement across different assessments.

**Table 4:** Multivariate logistic regression analysis of risk factors for hemorrhagic transformation.

Predictors	Hemorrhagic transformation		
	OR	CI	*p*-Value
ICVs asymmetry	3.809	1.582–9.171	0.003^*^
Presentation NIHSS score	0.989	0.929–1.052	0.717
ASPECTS	0.771	0.608–0.978	0.032^*^
IV-rtPA	2.847	1.098–7.385	0.031^*^
Location of vessel occlusion			
Internal carotid artery	–	–	–
MCA M1 segment	0.772	0.301–1.982	0.591
MCA M2 segment	0.561	0.090–3.489	0.536
Poor collateral (ASITN/SIR <3)	3.998	1.572–10.169	0.004^*^

^*^p < 0.05 indicates statistical significance.

ASPECTS, Alberta stroke program early CT score; ASITN/SIR, American Society of Interventional and Therapeutic Neuroradiology/Society of Interventional Radiology; HT, Hemorrhagic Transformation; ICVs, internal cerebral Veins; IV-rtPA, intravenous recombinant tissue plasminogen activator; MCA, middle cerebral artery; NIHSS, National Institutes of Health Stroke Scale.

## 4 Discussion

HT is a main side effect of EVT, making the identification of predictors crucial for improving patient outcomes. According to the results of our study, ICV asymmetry on baseline CTA can independently predict HT in patients with anterior circulation AIS-LVO after EVT. Moreover, ICV asymmetry is associated with sICH, which is a severe and life-altering complication.

HT after recanalization therapy in acute ischemic stroke is a severe complication associated with high rates of disability and mortality, especially due to parenchymal hematoma (PH) or sICH (13). Clinical features accompanied by an increased risk of HT in AIS-LVO patients include treatment with IV-rtPA, baseline NIHSS scores, glucose levels, atrial fibrillation, and poor collateral circulation (14). Our study is a noteworthy finding that adds to previous studies, which showed that ICV asymmetry is an independent predictor of poor functional outcomes after intravenous thrombolysis (7) and EVT (8). Interestingly, asymmetrical prominent cortical vein signs can also predict early neurological deterioration in acute ischemic stroke patients (15). This highlights the significance of retrograde venous flow in predicting clinical outcomes in AIS.

The ICVs drain the deep cerebral white matter, including regions such as the thalamus, basal ganglia, hippocampus, and medial temporal lobe, through the thalamostriate and anterior septal veins. These veins receive their arterial supply from the lateral lenticulostriate artery, medial lenticulostriate artery, and anterior choroidal artery. This extensive drainage network makes the ICVs an effective surrogate imaging biomarker for assessing cerebral perfusion in brain tissue. Although quantitative metrics, such as HU values, could enhance the precision of ICV asymmetry assessment, this study utilized qualitative visual assessments due to the limitations of imaging protocols. This method aligns with real-world clinical practice and demonstrates substantial interrater reliability. Hypo-opacification in the ipsilateral ICV reflects hypoperfusion in the cerebral hemisphere and is associated with an increased risk of HT. This finding aligns with prior studies showing that hypoperfusion detected by MRI predicts HT in acute ischemic stroke (16).

Several other studies (17, 18) used the cortical vein opacification score (COVES) to predict prognosis in AIS-LVO patients who underwent EVT. The COVES quantifies venous opacification on single-phase CTA of the vein of Labbé, sphenoparietal sinus, and superficial middle cerebral was used to determine venous outflow (VO) profiles. However, those superficial venous systems are more prone to variation than the deep venous system and are more difficult to identify. Conversely, the ICVs are located close to each other for effortless parallel comparison. Our study’s high degree of consistency for ICV asymmetry (*κ* = 0.86) shows that this is an easy assessment method. Therefore, using this robust technique, our study showed that ICV asymmetry independently predicts HT in patients with AIS-LVO. However, the reasons for the association between ICV asymmetry and HT were not totally demonstrated. We hypothesized that the poor ICV flow is a hallmark of hypoperfusion in the microcirculation, leading to the transformation of the ischemic penumbra to a huge cerebral infarction. When vessel occlusion occurs, the integrity of the blood–brain barrier (BBB) will be disrupted with the extension of ischemic time, leading to the extravasation of red blood cells from the blood vessels, resulting in HT. However, EVT was performed within the recommended time window.

According to the results of our study, other predictors for HT were poor collateral circulation, baseline ASPECTS, and IV-tPA. Previous studies have stated that good collateral circulation could diminish the severity of neurological symptoms and improve clinical outcomes (19, 20). Our study found that patients with poor collateral circulation had a higher risk of HT after receiving EVT. Interestingly, we observed that ICV asymmetry was often associated with poor collateral circulation on DSA (digital subtraction angiography), although these data were not explicitly shown. However, it is remarkable that a significant number of AIS-LVO patients developed ICV asymmetry despite good collateral circulation (18), possibly owing to damaged autoregulation or tissue micro-perfusion that hampers cerebral blood flow (CBF) on a tissue level (21).

Additionally, individual anatomical variations in venous drainage pathways may also contribute to ICV asymmetry. For instance, asymmetry in the size, structure, or dominance of internal cerebral veins (ICVs) could result in differential venous drainage patterns even in the presence of adequate collateral circulation. Variations in venous collateral pathways or compensatory shifts in drainage dynamics due to ischemia might further exacerbate this asymmetry. This may explain the missing connection between ICV asymmetry, arterial collateral status, and HT in this study. In addition, an increasing number of research studies are needed to elucidate the underlying pathophysiological mechanisms.

Lower ASPECTS, indicating a larger infarct core, has been consistently associated with HT risk due to more severe ischemic damage and loss of vascular integrity (3, 22). The robust association between baseline ASPECTS and HT in this study reconfirmed the previous research and further identified the infarct volume as one of the most significant factors influencing HT after EVT. IV-rtPA is another variable that is certain and has been established to be associated with HT. The coagulopathy and immune invasion of the neurovascular unit induced by the tPA exacerbate HT risk after reperfusion therapy (23, 24). Unlike IV-rtPA and ASPECTS, which predominantly reflect arterial mechanisms or the extent of ischemic burden, ICV asymmetry provides unique insights into delayed venous outflow and microvascular hemodynamics, both of which may contribute to an increased risk of HT. Our findings build upon previous research by highlighting ICV asymmetry as an independent risk factor for HT, offering a novel perspective on venous involvement in HT pathogenesis.

We recognized that our study has several limitations. First, this study had a small sample size and was a single-center retrospective cohort study. Second, we used visual inspection to assess ICV asymmetry, and any anatomical variation in the ICVs could potentially bias the results. Additionally, the number of variables included in the multivariate analysis may have been excessive for the small cohort size, possibly leading to issues such as overfitting. This discrepancy should be mentioned in the limitations of our study to ensure readers understand the potential impact on our findings.

## 5 Conclusion

Our study indicates that ICV asymmetry could independently predict HT and is associated with sICH in patients with anterior circulation AIS-LVO after EVT. Due to the relatively small size of our cohort, our study results should be cautiously interpreted.

## Data Availability

The raw data supporting the conclusions of this article will be made available by the authors without undue reservation.
